# Whole-genome sequencing identifies I-SceI-mediated transgene integration sites in *Xenopus tropicalis snai2:eGFP* line

**DOI:** 10.1093/g3journal/jkac037

**Published:** 2022-02-16

**Authors:** Jian Wang, Congyu Lu, Shuo Wei

**Affiliations:** 1 Department of Biological Sciences and Center for Bioinformatics and Computational Biology, University of Delaware, Newark, DE 19716, USA; 2 Department of Computational Biology, St. Jude Children’s Research Hospital, Memphis, TN 38105, USA

**Keywords:** whole-genome sequencing, transgenesis, I-SceI, *Xenopus tropicalis*, *snai2:eGFP* line

## Abstract

Transgenesis with the meganuclease I-SceI is a safe and efficient method, but the underlying mechanisms remain unclear due to the lack of information on transgene localization. Using I-SceI, we previously developed a transgenic *Xenopus tropicalis* line expressing enhanced green fluorescent protein driven by the neural crest-specific *snai2* promoter/enhancer, which is a powerful tool for studying neural crest development and craniofacial morphogenesis. Here, we carried out whole-genome shotgun sequencing for the *snai2:eGFP* embryos to identify the transgene integration sites. With a 19x sequencing coverage, we estimated that 6 copies of the transgene were inserted into the *Xenopus tropicalis* genome in the hemizygous transgenic embryos. Two transgene integration loci adjacent to each other were identified in a noncoding region on chromosome 1, possibly as a result of duplication after a single transgene insertion. Interestingly, genomic DNA at the boundaries of the transgene integration loci contains short sequences homologous to the I-SceI recognition site, suggesting that the integration was not random but probably mediated by sequence homology. To our knowledge, our work represents the first genome-wide sequencing study on a transgenic organism generated with I-SceI, which is useful for evaluating the potential genetic effects of I-SceI-mediated transgenesis and further understanding the mechanisms underlying this transgenic method.

## Introduction 

I-SceI is a meganuclease originally identified in *Saccharomyces cerevisiae* and recognizes an 18-bp nonpalindromic sequence (Monteilhet *et al.* 1990; Plessis *et al.* 1992). This enzyme can induce high-efficiency transgenesis in various species, ranging from ascidians to vertebrates such as fishes, amphibians and mammals, without the aid of special techniques or equipment (Thermes *et al.* 2002; Deschet *et al.* 2003; Ogino *et al.* 2006b; Pan *et al.* 2006; Wang *et al.* 2014). I-SceI-mediated transgenesis is also thought to be of less biosafety concern than the virus- or transposon-mediated approaches, as the transgenic elements are not supposed to be mobile (Wang *et al.* 2014). In *Xenopus*, I-SceI-mediated transgenesis is technically straightforward. Briefly, a DNA construct with the transgene cassette flanked by 2 I-SceI recognition sites in opposite orientations is digested by the I-SceI enzyme, and the whole reaction mixture is injected into fertilized *Xenopus* eggs (Ogino *et al.* 2006a; Ishibashi *et al.* 2012). This simple approach can result in ∼30% transgenesis efficiency in *Xenopus tropicalis* and ∼20% in *Xenopus laevis*, with relatively small copy number (typically 1–4) of the transgene cassette inserted into 1 or 2 loci of the target genome (Ogino *et al.* 2006a, 2006b). However, despite the success of this method, the underlying molecular mechanisms are still elusive, as there is little information on where the transgenes were inserted into the host genomes. This information is also important for assessing whether the transgene insertions may disrupt nearby genes and affect the development and health of the host, and for planning future crosses with other genetically modified animals.

Various techniques, including polymerase chain reaction (PCR)-based chromosome walking (Triglia *et al.* 1988; Yuanxin *et al.* 2003), inverse PCR (Cooley *et al.* 1988), fluorescence in situ hybridization (FISH) (Abrahams *et al.* 2003), and southern blotting (Guttikonda *et al.* 2016), have been used to characterize transgenic events. However, these techniques are either technically challenging or not very informative. For example, despite being commonly used for characterizing inserted DNA for many years, chromosome walking and inverse PCR are limited by the availability of optimal restriction sites for digesting the inserted fragments (Cooley *et al.* 1988). Southern blotting alone cannot be used to localize the insertion site. Although southern blotting has been coupled with Sanger sequencing to characterize the transgenic organisms, it is both time and resource consuming. FISH has been implemented for many years to identify the insertion of DNA fragments into specific chromosomes (Moscone *et al.* 1996; Nakanishi *et al.* 2002); however, it confers no information about the surrounding sequence of the insertion site. Furthermore, all PCR- and Sanger sequencing-based methods are limited by the insertion size, and sequencing complex regions of the genomes remains a challenge (Cosart *et al.* 2011). In contrast, next-generation sequencing (NGS) has emerged as a rapid and cost-effective option for identifying and characterizing transgene integrations. Successful identification of transgene insertion sites using paired-end high-throughput sequencing has been reported (Zhang *et al.* 2012; Park *et al.* 2017). Thus, NGS provides a powerful tool for genome-wide characterization of transgenic events.

Recently, we generated a transgenic *X. tropicalis* line expressing enhanced green fluorescent protein (eGFP) driven by the *snai2* promoter/enhancer (the *snai2:eGFP* line), which can be used for live imaging of neural crest development (Li *et al.* 2019). To identify and characterize the transgene integration sites, we carried out paired-end whole-genome sequencing (WGS) of hemizygous *snai2:eGFP* transgenic embryos. Our results provide the much needed genome-wide sequence information, which is valuable for assessing the potential effects of the transgene insertions on normal development and health of the *snai2:eGFP* transgenic frogs, and for understanding the molecular mechanisms underlying I-SceI-mediated transgenesis.

## Materials and methods

### Transgenic animals and genomic DNA preparation

The *snai2:eGFP* *X.* *tropicalis* line was generated using the I-SceI-mediated transgenesis in a previous study (Li *et al.* 2019). Hemizygous *snai2:eGFP* transgenic frogs were crossed with wild-type *X. tropicalis* frogs, and hemizygous transgenic embryos were selected by eGFP expression at stage ∼19. Transgenic embryos were stored at −80°C, and genomic DNA was prepared as described (Sive *et al.* 2000).

### WGS of the *snai2:eGFP* genome

WGS was performed using the Illumina HiSeq1500 sequencing system (Illumina Inc.). Short-insert paired-end genomic DNA library generation was conducted at Marshall University Genomics Core Facility using the TruSeq sample preparation kit (Illumina). Briefly, 2.0 μg of genomic DNA was sheared and size-selected to obtain fragments of ∼350 bp after quality control analysis. The genomic DNA library generated was sequenced in 2 × 101 bp paired-end mode with the Illumina HiSeq 1500 system. Low-quality reads were filtered out. Quality control for the reads was conducted with FastQC (https://www.bioinformatics.babraham.ac.uk/projects/fastqc/; accessed 2022 Feb 28), which showed that the average read quality scores were greater than 30.

### Assessment of transgene copy number

The number of reads mapped to a region follows the Poisson distribution and is expected to be proportional to the number of times the region appears in the DNA sample (Magi *et al.* 2011). In this study, the reads from WGS were mapped to the reference genome (James-Zorn *et al.* 2018; Karimi *et al.* 2018) and transgene sequence, respectively, using Bowtie2 (Langmead and Salzberg 2012). GC correction was then performed to reduce the impact of GC-bias using correctGCBias (Benjamini and Speed 2012; Ramírez *et al.* 2014). Copy number was estimated by comparing the coverage of the reference genome to the transgene coverage. ProteinPaint (Zhou *et al.* 2016) was used to visualize the read coverage.

### Identification of the transgene integration loci

**Fig. 1.**
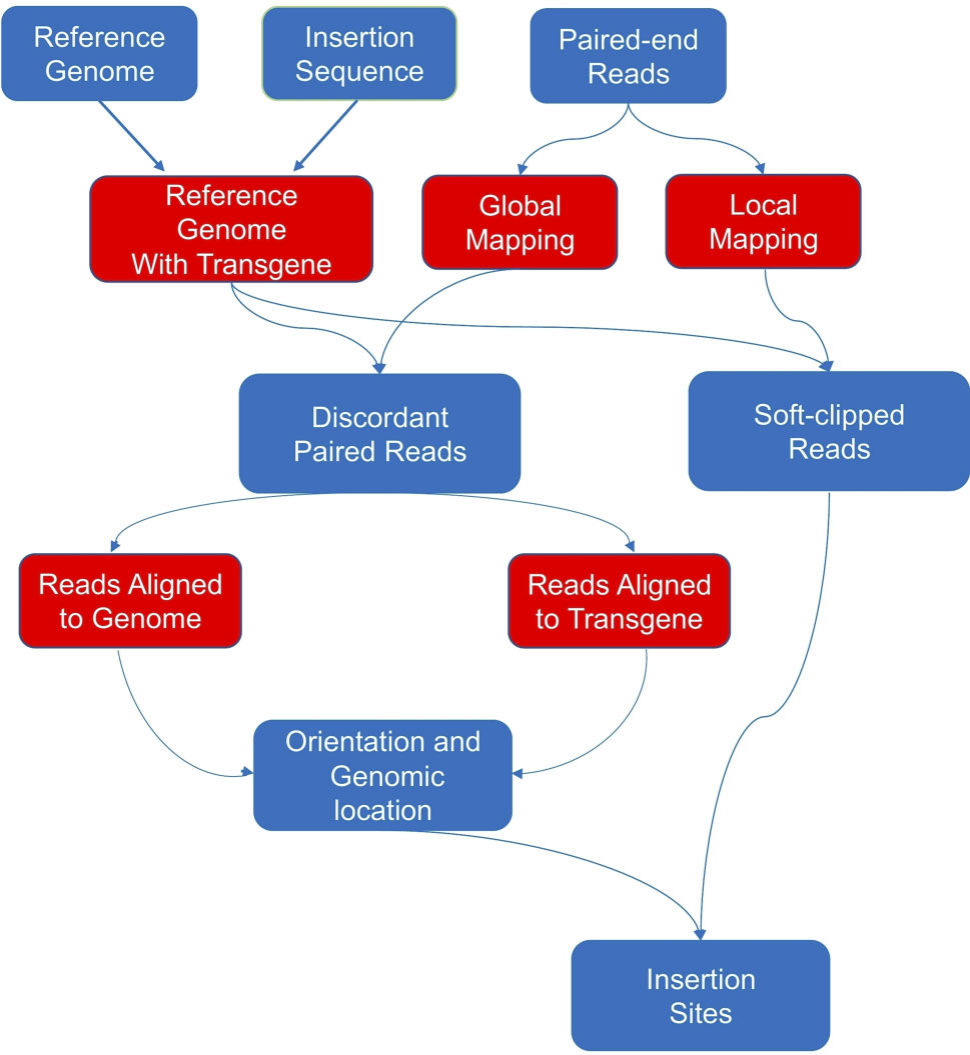
Workflow for detection of integration sites. Workflow detailing each major step in the pipeline of detection of transgene integration sites in the *X. tropicalis* genome.

The transgene cassette includes ∼3,900 bp endogenous *snai2* promoter sequence followed by *eGFP* sequence with the length of ∼700 bp, which was inserted into the ∼3 kb transgenic vector ISceI-pBSII-SK+ (Supplementary Table 1) (Ogino *et al.* 2006a). The transgene sequence was added to the reference genome of *X. tropicalis* as an extra chromosome. The combined sequences were used for bowtie2 index building. Paired sequence reads from WGS were mapped to the constructed reference twice using Bowtie2 (Langmead and Salzberg 2012) in global (reads align end to end) and local modes (reads are soft clipped), separately (Fig. 1). From the global mapping, only uniquely and discordantly mapped read pairs where 1 read was mapped to the *snai2* promoter region or extra chromosome and its mate was mapped to other location on genome were retained for identification of the integration sites. The reads from local alignment mode were “softclipped” at 1 or both ends to optimize alignment score. This approach identified several “split reads,” which had either ends mapped to integration location and enabled the detection of integration sites at base pair resolution.

### Validation of the insertion by PCR

To validate the integration sites, 4 pairs of primers were designed to generate amplicons that spans all boundary regions (Fig. 4a and Supplementary Table 2). All primers were designed based on the reads mapped to insertion region. The first pair of primers covered the left boundary of integration site 1, the second pair amplified the right boundary of integration site 2, the third pair covered the left boundary of integration site 2, and the last pair covered the entire region between integration sites 1 and 2.

## Results

### WGS of the *snai2:eGFP* transgenic *X. tropicalis* embryos

Using the I-SceI meganuclease, we recently generated the *snai2:eGFP* transgenic *X. tropicalis* line for live imaging of neural crest development (Li *et al.* 2019). To evaluate the potential effects of transgene insertions on the normal development and health of *X. tropicalis*, and to study the mechanisms of I-SceI-mediated transgenesis, it is important to characterize the transgenic line at the molecular level. To this end, Illumina WGS of the *snai2:eGFP* transgenic embryos was carried out on paired-end library, and 276,284,290 clean reads were generated after quality control. From the global mapping result, 95.52% of the clean reads were mapped to the *X. tropicalis* 9.1 reference genome sequence, representing 19x genome coverage (Table 1). The transgene construct in the present study (GenBank accession number OM212390) is approximately 8 kb long, including an endogenous *snai2* promoter/enhancer (hereinafter referred to as *snai2* promoter), the eGFP coding sequence, and the ISceI-pBSII-SK+ transgenic vector. The information of the integrated sequence was used to streamline our analysis strategy and has facilitated the detection of the integration loci.

**Table 1.** Summary of WGS results and orphaned read mapping.

Categories	WGS (paired-end library)
Total number of reads	276,711,936
Quality filtered reads	276,284,290
Mapping ration	95.52%
Sequencing coverage	19×
Reads found in the candidate insertion site region	32

### Estimation of the copy number of the transgene cassette in hemizygous *snai2:eGFP* embryos

**Fig. 2.**
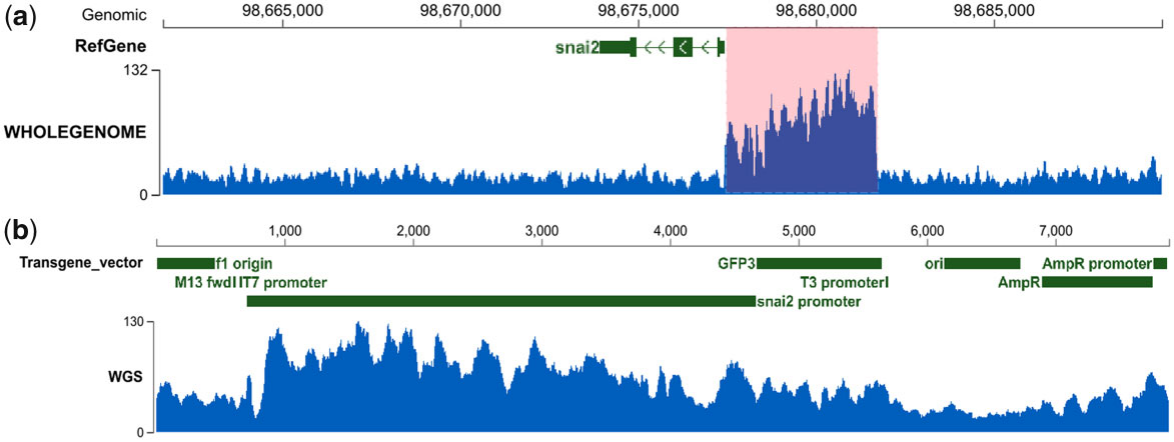
Sequencing depth of WGS reads mapped to the *snai2* gene and transgene sequences. a) Sequencing depth of the *snai2* gene and surrounding region on chromosome 1. The *snai2* promoter, which is also part of the transgene construct, is highlighted in pink, and the transcribed region is shown in green. b) Sequencing depth of different regions of the transgene. Reads with low mapping quality, including those with low complexity and those perfectly mapped to other regions of the genome, have been removed. *Y*-axis represents sequencing coverage.

To determine the copy number of the transgene inserted into the *X. tropicalis* genome, WGS reads were mapped to reference genome and the transgene sequence, respectively. The mapped BAM files were converted to coverage track files (bigWig) after the GC-Bias correction (Kent *et al.* 2010; Talevich *et al.* 2016). The visualization of reads covering the *snai2* promoter (from both the endogenous genome and the transgene construct) and the transgene construct sequence is shown in Fig. 2.

The copy number of transgene was estimated by comparing the coverage of the *snai2* promoter with the *X. tropicalis* genome. As shown in Fig. 2a, the coverage for the transcribed region of *snai2* gene and the rest of the reference genome was around 20x, while the coverage for *snai2* promoter (highlighted in Fig. 2a) was 80x, suggesting that 6 copies of *snai2* promoter sequence was from the transgene. In addition, the sequencing coverage for the *eGFP* (*GFP3*) coding sequence was around 60x, also indicating 6 copies of *eGFP* insertion (Fig. 2b). In conclusion, we estimate that ∼6 copies of transgene cassette were inserted into the hemizygous *snai2:eGFP* embryos.

### The *snai2:eGFP* transgene was identified in 2 adjacent loci in *X. tropicalis* Chromosome 1

**Fig. 3.**
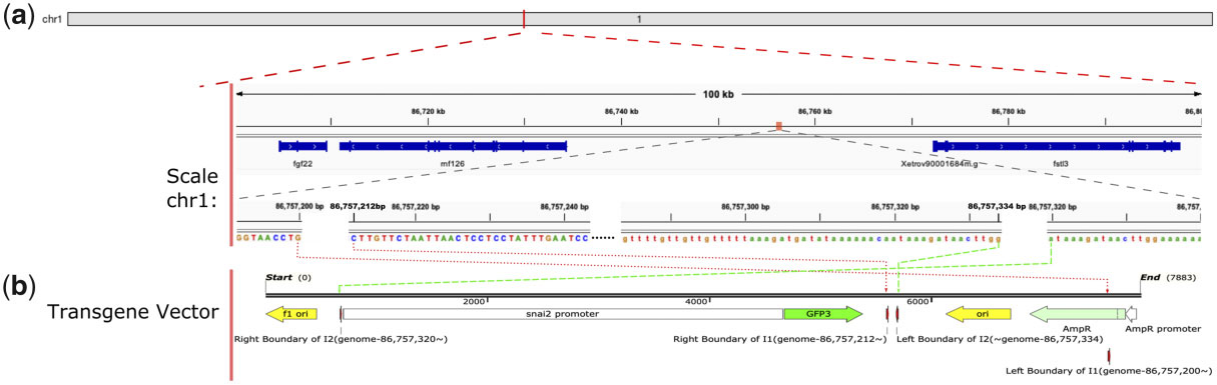
Mapping of the 2 transgene integration loci to chromosome 1 of the *X. tropicalis* genome. a) Localization of the transgene insertions in chromosome 1. The 100-kb surrounding region (middle) includes the nearby genes, and the insertion region is further zoomed in to show the locations of the 2 transgene integration sites and the genomic DNA sequences at the boundaries (bottom). b) A linear map of the transgene construct. The genomic DNA and transgene sequences are connected with red and green dotted arrows, respectively, at the 2 integration sites (I1 and I2). See Supplementary Fig. 1 for WGS reads that align with the junctions of transgene integration sites. Junction sequences were further confirmed by PCR and Sanger sequencing (see Fig. 4).

The WGS generated a mixture of 3 types of fragments: those derived solely from the *X. tropicalis* genome, those derived solely from the transgene, and those derived from regions spanning the transgene integration sites. When mapped back to the reference genome and transgene sequences, the paired-end reads from the third type of fragments will have 1 read of a pair mapped to the *X. tropicalis* genome and its mate mapped to the transgene. This class of paired-end reads are referred to as “junction pairs” here. To pinpoint the location of transgene integration, paired-end reads were mapped to reference genome and the transgene sequence using Bowtie2 in a “local” alignment mode to detect the junction pairs. As shown in Supplementary Fig. 1, by performing “soft-clip” at 1 or both ends of reads to optimize alignment score and allow for local alignment, we detected 17 reads spanning either the 5′ or the 3′ junction of the integration sites. Out of these, 8 soft-clipped reads were mapped to 1 genomic location in chromosome 1, and an additional 9 were mapped to a nearby genomic location (Fig. 3, a and b and Supplementary Fig. 1). These 2 integration sites are referred to as integration site 1 (I1) and integration site 2 (I2) hereinafter. The left boundary of I1 was mapped to position 86,757,200 of chromosome 1, and the right boundary was located at position 86,757,212 of the same chromosome; the left and right boundaries of I2 were mapped to position 86,757,334 and 86,757,320, respectively. Thus, the 2 integration sites are merely 108 bp apart from each other. A well-known characteristic signature of transgene integration is that this process normally causes genomic DNA rearrangement, including deletion, duplication and translocation. In the current study, a deletion of an 11-bp portion (86,757,201–86,757,211) of the *X. tropicalis* genome at I1 was observed. In addition, a 15-bp genomic DNA duplication (86,757,320–86757334) was detected at I2 (Fig. 3, a and b and Supplementary Fig. 1). Both integration sites are located within an intergenic region. The nearest gene from upstream side is *rnf126* and located at 25 kb away from I1, while the nearest downstream gene is *fstl3*, which is more than 12 kb away (Fig. 3a). Thus, it is unlikely that the transgene insertions will have any severe effects on the expression of nearby genes. We have also identified 3 boundaries between 2 different copies of the transgene construct, as supported by paired-end reads (Supplementary Fig. 2), consistent with previous finding that a few copies of transgene form concatemers when inserted into the *X. tropicalis* genome (Ogino *et al.* 2006a, 2006b).

**Fig. 4.**
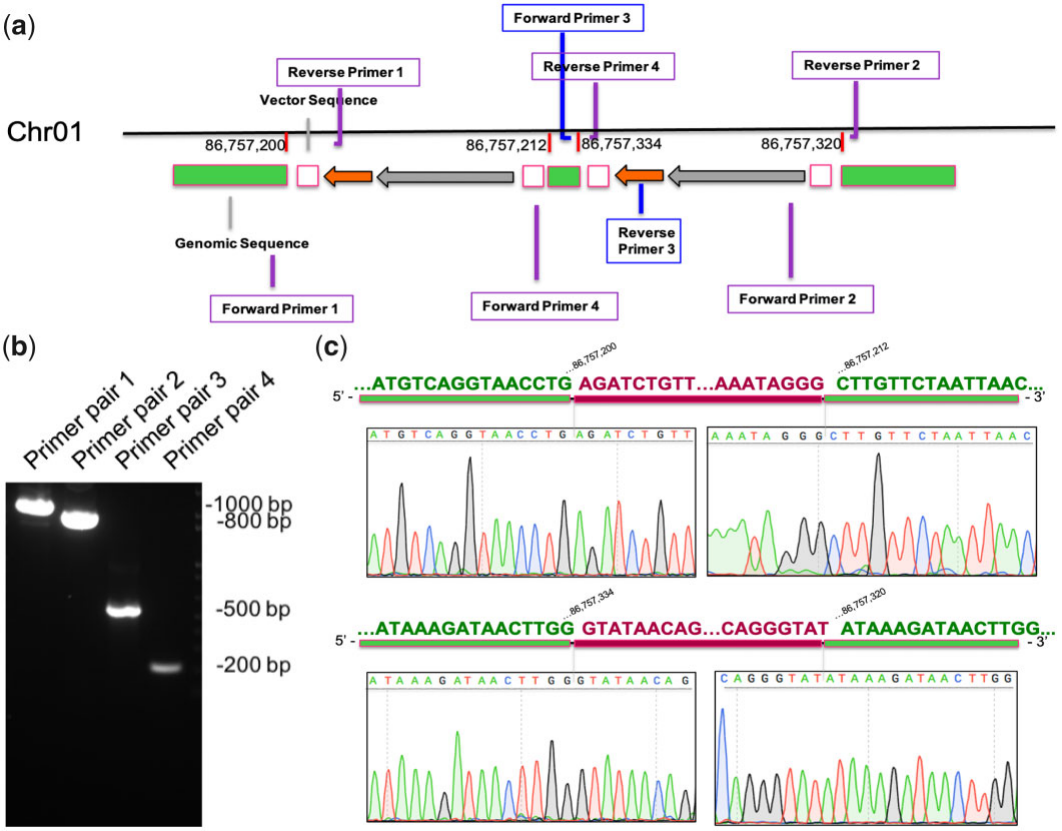
Locations of primers and PCR validation of transgene integration sites. a) Design of PCR primers. Primer pairs 1 and 3 covered the left boundaries of the transgene integration sites (I1 and I2), primer pair 2 covered the right boundary of I2, and primer pair 4 covered the region between I1 and I2 along with the flanking transgene sequences. Green bars represent *Xenopus* genomic DNA, gray and orange arrows represent the *snai2* promoter and eGFP coding sequence from the transgene, respectively, and white blocks represent the vector sequences from the transgene. Segments are separated from each other for better visualization. b) PCR amplifications of junction sequences using the primers shown in (a). c) Sanger sequencing results across the insertion boundaries of I1 (upper) and I2 (lower).

To confirm the transgene integration sites, we carried out PCR reactions to amplify the junctions between the transgene and chromosome, as well as Sanger sequencing for the PCR products. Junctions of I2 were amplified for both the upstream and downstream border sequences using primer pairs 3 and 2 (Fig. 4a), generating products of ∼500 bases and ∼800 bases, respectively (Fig. 4b). The results from Sanger sequencing are consistent with WGS data, confirming the integration site detection (compare the lower panel of Fig. 4c with Supplementary Fig. 1, c and d). Primer pair 1 amplified the upstream boundary of I1 to generate a PCR product of ∼1,000 bp, and the ∼200 bp amplicon generated by primer pair 4 covered the entire genomic region between 2 insertion sites and the flanking transgene sequences (Fig. 4, a and b). Again, Sanger sequencing results validated both the downstream and downstream boundaries of I1 (compare the upper panel of Fig. 4c with Supplementary Fig. 1, a and b).

**Fig. 5.**
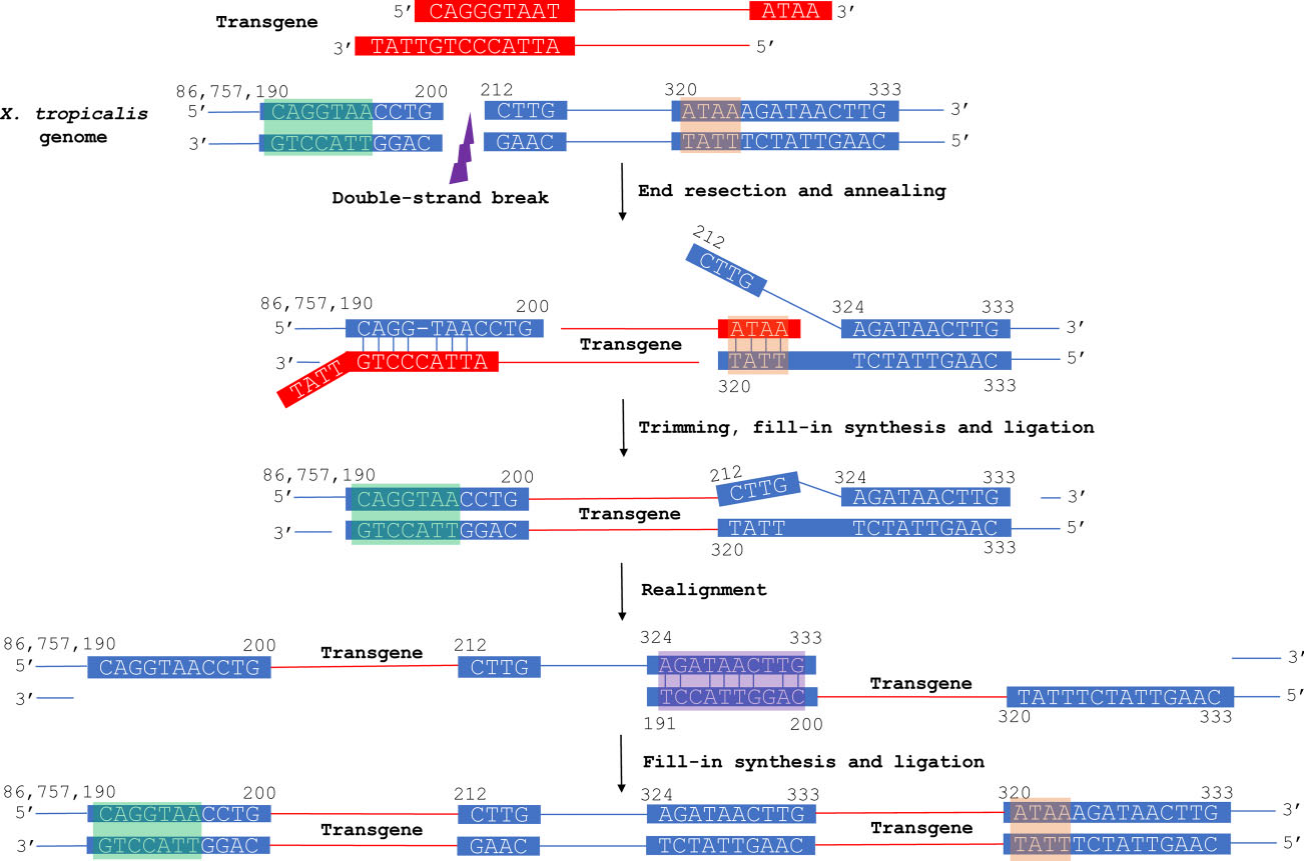
A model for I-SceI-mediated transgenesis that resulted in the *snai2:eGFP X. tropicalis* line. Genomic DNA is shown in blue and transgene construct in red. Green and orange boxes highlight the sequence homology between the genomic DNA at the junctions and the I-SceI recognition sequence at the left end of transgene construct and the 3′-overhang generated by I-SceI cleavage at the right end, respectively. Purple box highlights the homology between the upstream and downstream genomic sequences at the initial transgene insertion site that possibly led to a subsequent realignment. See *Discussion* for further explanation.

Finally, to test if the transgene integrations occur randomly or have certain preferences, we analyzed the genomic sequences at the boundaries of transgene insertions (Fig. 4c and Supplementary Fig. 1). Interestingly, the 4-bp genomic sequence downstream of I2 (5′-ATAA-3′) is identical to the 3′-overhang generated by I-SceI digestion (highlighted in orange boxes in Fig. 5), whereas the genomic sequence upstream of I1 also contains a short sequence homologous to the I-SceI recognition site (green boxes in Fig. 5). We further identified partial homology between the sequences upstream of I1 and I2 (purple box in Fig. 5), which might mediate the realignment of the genome and duplication of the originally inserted transgene sequence (Fig. 5). Thus, the transgene integration is probably not random but mediated by the homology between the host genome and I-SceI recognition sequence (see *Discussion*).

## Discussion

Endonucleases are widely used for transgenesis, primarily by causing double-strand breaks and stimulating recombination to facilitate the integration of transgene constructs (Chesneau *et al.* 2008; Grabher and Wittbrodt 2008; Ishibashi *et al.* 2012). The meganuclease I-SceI has been shown to successfully mediate transgenesis in several animal species (Thermes *et al.* 2002; Deschet *et al.* 2003; Pan *et al.* 2006; Ogino *et al.* 2006b; Wang *et al.* 2014). However, the mechanisms underlying I-SceI-mediated transgenesis remain unclear, and to our knowledge the transgene integration sites have not been determined for any of the transgenic animals generated using this method.

We recently used I-SceI to generate the *snai2:eGFP* transgenic *X. tropicalis* line for live imaging of neural crest development (Li *et al.* 2019). Although the transgenic frogs appear healthy and develop normally (Li *et al.* 2019), detailed information on the transgene insertion locations is still needed to evaluate if these insertions disrupt the nearby nonessential genes. Such information is also important for planning crosses with other genetically modified animals, to ensure that the alleles to be combined are not on the same chromosome and can be properly segregated. To this end, we carried out WGS for the genomic DNA extracted from hemizygous *snai2:eGFP* transgenic embryos with a 19x coverage. Based on the sequencing results, we mapped the transgene insertions to a noncoding region on chromosome 1 far away from the closest genes (Fig. 3a), confirming that these insertions are unlikely to cause any detrimental effects to the host. By calculating the sequence coverage, we estimate that 6 copies of the transgene cassette were inserted into the host genome, consistent with previous reports that this method typically causes insertions of a small copy number of transgenes (Thermes *et al.* 2002; Ishibashi *et al.* 2012). These are in contrast to the more traditional transgenic methods such as restriction endonuclease-mediated integration (REMI), which often lead to the formation of long transgene concatemers inserted into multiple regions in the host genome (Chesneau *et al.* 2008).

Chromosome breaks, occurring either spontaneously or by exogenous restriction endonuclease-mediated cleavage, are believed to create the sites for integration of linear DNA during transgenesis (Brinster *et al.* 1985; Chesneau *et al.* 2008). The meganuclease I-SceI recognizes an 18-bp nonpalindromic target sequence, which occurs only once every 70 Gbp of random sequence in theory. Consequently, no endogenous I-SceI recognition site has been identified in any animal species, including *X. tropicalis*, leading to the speculation that I-SceI-mediated transgenesis does not require cutting of the target genome (Ogino *et al.* 2006a; Grabher and Wittbrodt 2008; Ishibashi *et al.* 2012). Although I-SceI has been shown to tolerate minimal degeneracy at the recognition sequence in vitro (Colleaux *et al.* 1988), there is no evidence suggesting that this happens during transgenesis. Therefore, the mechanisms of I-SceI-meditated transgenesis are likely different from those of the classic REMI method, in which the co-injected restriction enzymes partially digest the host chromosomes to stimulate transgene integration (Ogino *et al.* 2006a). Instead, a preexisting double-strand break in the host genome is probably needed to allow the transgenes to be integrated in this case.

If I-SceI is not required for generating chromosome breaks, how does it promote transgenesis, then? It has been proposed that in the injected reaction mixture, I-SceI continues to bind to the digested ends of the transgenic construct to protect the linearized plasmid from degradation. This stable binding may also prevent the formation of long concatemers, which can cause silencing of transgene expression (Thermes *et al.* 2002). However, it is unclear if the I-SceI recognition sequences at the ends of the linearized transgene construct, especially the 4-nucleotide 3′-overhang generated by I-SceI digestion, plays any roles in the selection of the genomic locations to integrate into. Our WGS results reveal short sequence homology between the genomic DNA at the integration boundaries and the I-SceI recognition sequence, including the 3′-overhang (green and orange boxes in Fig. 5). Thus, it is likely that these transgene integration events are not random but mediated by short sequence homologies. Microhomology-mediated end joining (MMEJ) is an error-prone mechanism that efficiently repairs double-strand breaks in eukaryotic cells, resulting in deletions in the flanking sequences and sometimes duplications of nearby genomic fragments (McVey and Lee 2008). The close proximity of the 2 transgene insertion loci, the duplication of a 15-bp genomic DNA (86,757,320–86757334), and the partial homology between the genomic sequences upstream of insertion sites 1 and 2 (purple box in Fig. 5), suggest that the 2 transgene insertions were the result of a tandem duplication. Based on our sequence analyses, we hypothesize that the I-SceI-mediated transgenesis in our *snai2:eGFP* line involved 2 steps, an initial transgene integration through MMEJ and a subsequent tandem duplication of the integrated transgene along with the adjoining 15-bp genomic DNA (Fig. 5). Following a double-strand break in a host chromosome (chromosome 1), both newly generated ends were resected until 2 short sequences, an upstream 1 homologous to the left end of the transgene (part of the I-SceI recognition sequence) and a downstream 1 homologous to the 3′-overhang generated by I-SceI (green and orange boxes in Fig. 5, respectively), were exposed to facilitate the MMEJ-mediated insertion of the transgene. After trimming, fill-in synthesis and ligation, the 2 strands were realigned using the partial homology between the genomic sequences at the upstream and downstream junctions (purple box in Fig. 5), resulting in the duplication of the transgene insertion.

With the considerably reduced cost, NGS has been increasingly applied to the characterization of transgenic events in the recent years (Zhang *et al.* 2012; Park *et al.* 2017; Goodwin *et al.* 2019; Nicholls *et al.* 2019; Wang *et al.* 2020). In this study, we performed WGS to achieve a 19x coverage of the genomic sequence of the hemizygous *snai2:eGFP* transgenic *X. tropicalis* embryos generated using I-SceI. The acquired sequence information allowed us to map the transgene integration loci and calculate the copy number of the transgene cassettes inserted into the host genome. In particular, we were able to identify 2 transgene integration loci 108 bp apart, as well as a short deletion and a short duplication of the genomic DNA at the individual integration loci, respectively. Such detailed genetic information would have been difficult to obtain with the traditional PCR-Sanger sequencing-based mapping methods, as the repeated transgene cassettes present a tremendous obstacle for Sanger sequencing. With this information, we propose a model for the transgenesis that resulted in the *snai2:eGFP* line (Fig. 5). It would be of interest to carry out similar studies for other transgenic animals generated using the same method, to test if these mechanisms apply generally to I-SceI-mediated transgenesis.

## Data availability

All WGS raw reads in this study have been deposited in the NCBI SRA database (accession number PRJNA716660), https://www.ncbi.nlm.nih.gov/sra/?term=PRJNA716660 (accessed 2022 Feb 28).

Supplemental material is available at *G3* online.

## Supplementary Material

jkac037_Supplementary_Figures

jkac037_Supplementary_Table_S1

jkac037_Supplementary_Table_S2
