# Supplementary material for: Whole-genome sequencing identifies I-SceI-mediated transgene integration sites in Xenopus tropicalis snai2:eGFP line
Source: G3 (Bethesda). 2022 Feb 16;12(5):jkac037. doi: 10.1093/g3journal/jkac037 (PMC9073676; doi:10.1093/g3journal/jkac037)
Supplement: jkac037_Supplementary_Figures [file jkac037_supplementary_figures.pdf]

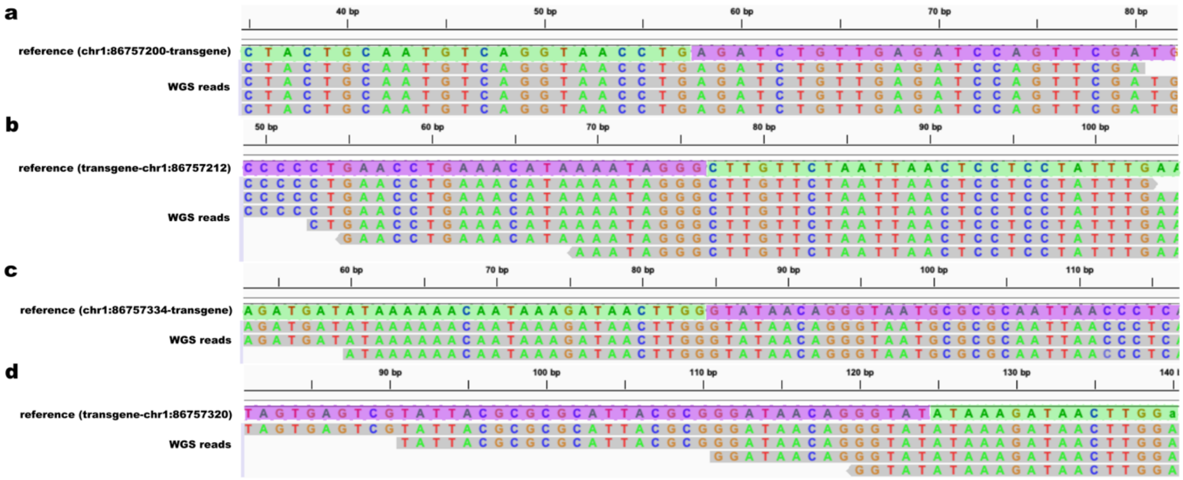

Figure S1. WGS reads mapped to the reconstructed Chromosome 1 with the inserted transgene. Soft-clipped reads were aligned to the left (a) and right (b) boundaries of transgene integration site 1 (I1), as well as the left (c) and right (d) boundaries of integration site 2 (I2). *X. tropicalis* genome, transgene sequence and WGS reads are highlighted with green, purple and gray, respectively.

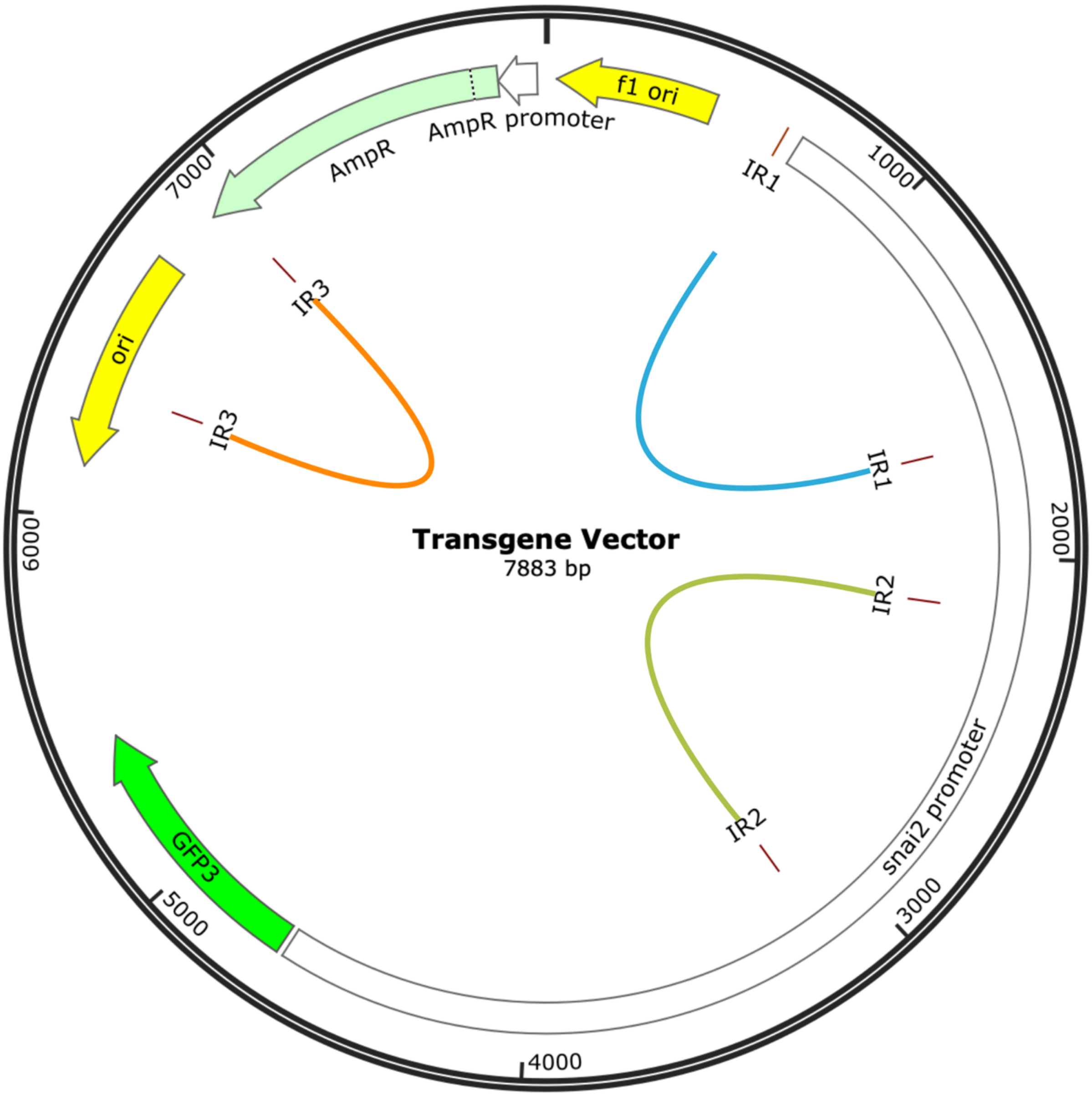

Figure S2. Recombination sites of transgene concatemer. Transgene construct was concatenated and inserted into genomic sequence on Chromosome 1. Three intramolecular recombination sites (IR1-3) were supported by WGS reads. The concatemeric joints between two copies are linked by curves of different colors.
